# Reevaluation of whether a Functional Agr-like Quorum-Sensing System Is Necessary for Production of Wild-Type Levels of Epsilon-Toxin by Clostridium perfringens Type D Strains

**DOI:** 10.1128/mbio.00496-22

**Published:** 2022-03-23

**Authors:** Iman Mehdizadeh Gohari, Jihong Li, Julian I. Rood, Bruce A. McClane

**Affiliations:** a Department of Microbiology and Molecular Genetics, University of Pittsburgh School of Medicine, Pittsburgh Pennsylvania, USA; b Infection Program, Monash Biomedicine Discovery Institute, Department of Microbiology, Monash University, Victoria, Australia; University of Delaware

## Abstract

Clostridium perfringens type B and D strains produce epsilon-toxin (ETX). Our 2011 *mBio* study (mBio 2:e00275-11, 2011, https://doi.org/10.1128/mBio.00275-11) reported that the Agr quorum-sensing (QS) system regulates ETX production by type D strain CN3718. However, subsequent studies have brought that conclusion into question. For example, we reported in 2012 (Infect Immun 80:3008–3017, 2012, https://doi.org/10.1128/IAI.00438-12) that the Agr-like QS system is not required for wild-type ETX production levels by two type B strains. Consequently, we reexamined whether the Agr-like QS system regulates ETX production in type D strains by using Targetron insertional mutagenesis to construct new *agrB* null mutants of two type D strains, CN3718 and CN2068. Western blotting showed that both *agrB* mutants still produce wild-type ETX levels. However, the newly constructed *agrB* mutants of both type D strains produced reduced amounts of alpha-toxin, and this effect was reversible by complementation, which confirms loss of functional AgrB production by these mutants since alpha-toxin production is known to be regulated by AgrB. Coupled with the previously published results for type B strains, these new findings indicate the Agr-like QS system is not usually necessary for C. perfringens to produce wild-type ETX levels.

## INTRODUCTION

In laboratory animal models and natural disease hosts, epsilon-toxin (ETX) plays a critical role in the virulence of Clostridium perfringens type D strains and, likely, type B strains (1–4). Our 2011 *mBio* paper (5) reported that an *agrB* null mutant of type D strain CN3718 grew similarly in tryptone-glucose-yeast extract (TGY) broth to its wild-type parent, but produced less ETX, and that this effect was partially reversible by complementation. Consequently, it was concluded that regulation of ETX production by CN3718 involves the Agr quorum-sensing (QS) system. In the same 2011 *mBio* paper, we also showed that a CN3718 *virS virR* null mutant still produces wild-type ETX levels.

Since the publication of that 2011 paper, the reliability of concluding that the Agr QS is necessary for production of wild-type ETX levels has come into question. Specifically, it has since been shown that (i) neither the Agr QS system nor the VirS VirR two-component regulatory system (TCRS) is required for production of wild-type ETX levels by two type B strains (6), (ii) production of all other C. perfringens toxins regulated by the Agr QS system also involves the VirS VirR TCRS (7–14), and (iii) the Agr QS signal peptide can bind directly to VirS as a receptor (15).

**FIG 1:**
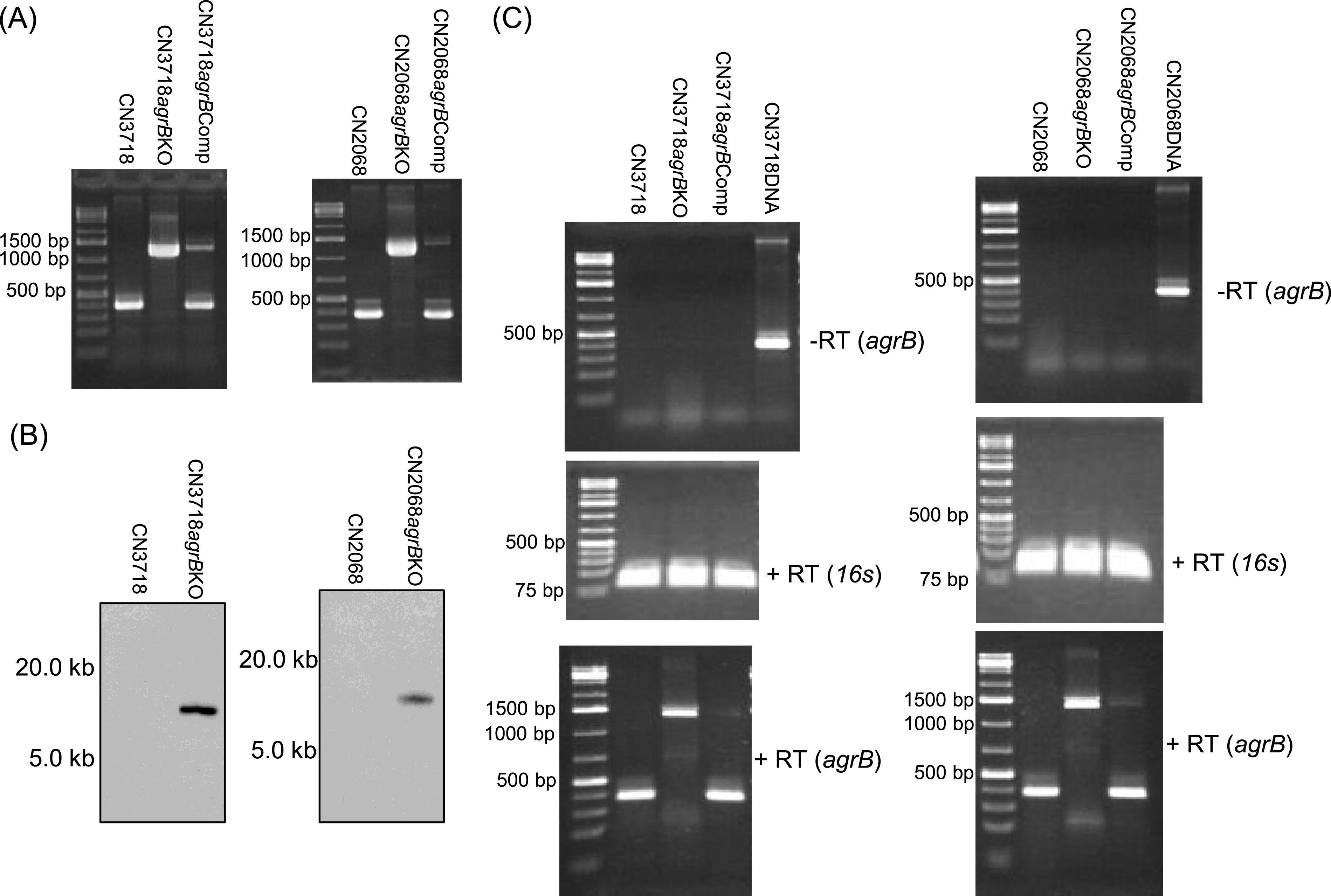
Construction and characterization of newly constructed *agrB* mutants of type D strains CN3718 and CN2068. (A) PCR confirmation of CN3718 and CN2068 *agrB* mutant strains. DNA purified from wild-type CN3718 or CN2068 supported amplification of a 366-bp product using internal *agrB* primers, while the same PCR assays amplified an ∼1.3-kb product using DNA purified from the mutant strains due to insertion of an ∼900-bp product into their *agrB* gene. (B) Southern blot hybridization of an intron-specific probe to DNA from CN3718 or CN2068 or their *agrB* mutants. DNA from each strain was digested with EcoRI and electrophoresed on a 1% agarose gel prior to blotting and hybridization with the intron-specific probe. (C) RT-PCR evaluation of *agrB* expression shows that the *agrB* mutants (left, CN3718 *agrB* KO; right, CN2068 *agrB* KO) expressed an intron::*agrB* fusion transcript, while the complementing strains (CN3718 *agrB* Comp and CN2068 *agrB* Comp) expressed the wild-type *agrB* transcript. These PCR assays were repeated three times, and a representative result is shown. For size reference, a 1-kb marker is shown (Fisher Scientific).

Due to those apparent discrepancies, we recently constructed a second *agrB* mutant and complementing strain in a different stock culture of CN3718 than that used in 2011 (Fig. 1A). This new *agrB* mutant was constructed by Targetron-mediated insertional mutagenesis (16) using the pJIR750agrBNi *agrB* knockout plasmid (17). A complementing strain was also constructed by electroporating the *agrB* complementation plasmid CPJVp3 (12) into the newly constructed CN3718 *agrB* mutant, as described previously (12).

**FIG 2:**
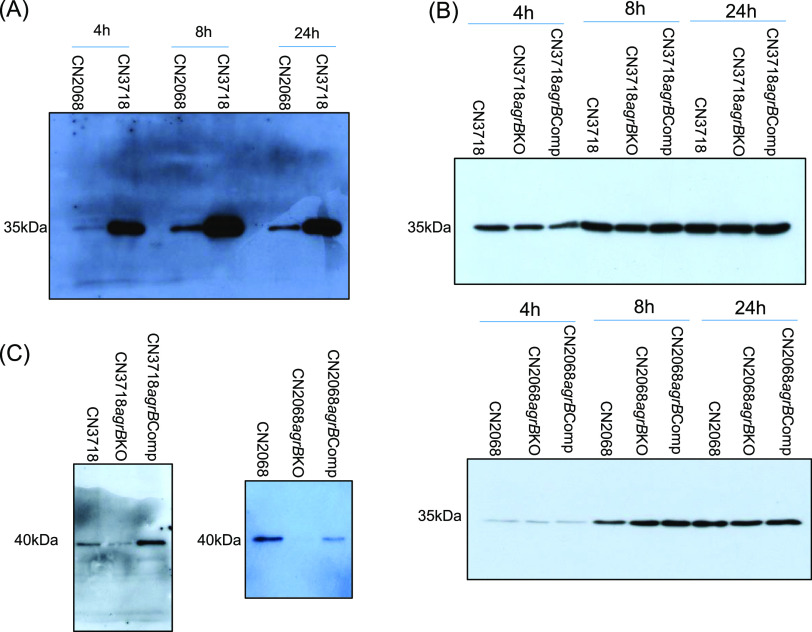
Phenotypic comparisons of Fig. 1 type D strains. Western blots showing (A) timeline of ETX production by wild-type CN3718 and CN2068, (B) a time point comparison of ETX production by wild-type CN3718 or CN2068 versus their *agrB* mutants (CN3718 *agrB* KO or CN2068 *agrB* KO) or complementing strains (CN3718 *agrB* Comp or CN2068 a*grB* Comp), and (C) CPA production by CN3718 and its derivatives (left) or CN2068 and its derivatives (right). All Western blot results shown in panels A to C are representative of three repetitions.

The characterization of this new CN3718 *agrB* mutant showed the presence of a single intron insertion (Fig. 1B), while reverse transcription-PCR (RT-PCR) demonstrated the presence of an intron::*agrB* fusion transcript (Fig. 1C). Under the same tryptone-glucose-yeast extract (TGY) broth culture conditions used in our 2011 *mBio* paper (5), no growth differences were measured between the wild-type parent and this *agrB* mutant (data not shown), and Western blotting using an ETX monoclonal antibody (5) confirmed that ETX was produced by wild-type CN3718 (Fig. 2A). Surprisingly, this Western blot analysis also revealed that, under these culture conditions, the *agrB* mutant still produced the same level of ETX as its wild-type parent or the complementing strain (Fig. 2B).

To confirm that introduction of an intron into the *agrB* gene in CN3718 had created an *agrB* null mutant, alpha-toxin (CPA) production by these cultures of CN3718 or its derivatives was also evaluated, since CPA production in C. perfringens is regulated by AgrB (12, 13). Consistent with the expected phenotype of an *agrB* null mutant, less CPA was produced by this mutant versus its parent, and this effect was reversible by complementation (Fig. 2C).

To further evaluate whether the Agr QS is necessary for production of wild-type ETX levels by type D strains, an *agrB* mutant and complementing strain were similarly constructed in CN2068 (18), a second type D strain (Fig. 1A). Southern blot analysis (Fig. 1B) demonstrated that the CN2068 *agrB* mutant contained only a single intron insertion. RT-PCR (Fig. 1C) showed that this CN2068 *agrB* mutant expressed an intron::*agrB* fusion transcript and that complementation had restored expression of the wild-type *agrB* transcript. Under the same culture conditions used in our 2011 *mBio* paper (5), no growth differences were noted between CN2068 and its *agrB* mutant (data not shown). Western blotting confirmed that, under these culture conditions, CN2068 produced ETX, although in smaller amounts than CN3718 (Fig. 2A). Western blotting also detected no differences in ETX production between the CN2068 *agrB* mutant and its wild-type parent or the complementing strain. In contrast, Western blots of the same cultures showed that this *agrB* null mutant produced much less CPA than wild-type CN2068 and that complementation substantially restored production of this toxin (Fig. 2C).

Coupling the new results presented above with our previous results indicating that inactivating the Agr QS does not affect ETX production levels by two type B strains (6), we conclude that the Agr QS is not usually necessary for type B or D strains to produce wild-type levels of ETX.
